# Enhanced Visible-Light Photocatalytic Activity of Bi-Doped ZnFe_2_O_4_ Thin Films toward Rhodamine B and Metronidazole Degradation

**DOI:** 10.1021/acsomega.6c03278

**Published:** 2026-09-28

**Authors:** Fatma Ezzahra Dhif, Neila Jebbari, Enrique Rodriguez Castellon, Pankaj Kumar, Alberto Vomiero, Elisa Moretti, Najoua Turki-Kamoun, Kassa Belay Ibrahim

**Affiliations:** † Laboratory of Condensed Matter Physics, Faculty of Sciences, 37964 University of Tunis El Manar, Tunis 2092, Tunisia; ‡ Departamento de Química Inorgánica, Cristalografía y Mineralogía, Facultad de Ciencias, Universidad de Málaga, Campus de Teatinos, Málaga 29071, Spain; § Department of Molecular Sciences and Nanosystems, 19047 Ca’ Foscari University of Venice, Via Torino 155, Venezia Mestre 30172, Italy; ∥ Division of Materials Science, Department of Engineering Sciences and Mathematics, 5185 Luleå University of Technology, Luleå SE-97187, Sweden

## Abstract

Bi-doped ZnFe_2_O_4_ thin films were fabricated via spray pyrolysis across a systematic doping series (0–0.8 at.% Bi) and characterized for their structural, morphological, optical, and photocatalytic properties. X-ray diffraction confirmed phase-pure ZnFe_2_O_4_ with enhanced crystallinity at concentrations of ≤ 0.4 at.% Bi, while a secondary BiFeO_3_ phase emerged at 0.6% and 0.8% Bi, marking the solubility limit of Bi within the host lattice. The 0.4% Bi-doped film exhibited the most refined microstructure, a homogeneous surface morphology, and uniform elemental distribution of Bi throughout the Bi-ZnFe_2_O_4_ matrix. Optical studies revealed a systematic band gap reduction to 2.12 eV at 0.4% Bi, significantly extending visible-light absorption. Under visible-light irradiation, this composition achieved the highest photocatalytic degradation efficiencies across the series, 89% for rhodamine B (RhB) and 75% for metronidazole (MDZ), within 120 min. These results establish Bi doping concentration as the decisive parameter governing structural integrity, optical response, and photocatalytic activity in ZnFe_2_O_4_ thin films, positioning the 0.4 at.% Bi composition as a highly promising candidate for efficient visible-light-driven photocatalytic water treatment.

## Graphical abstract

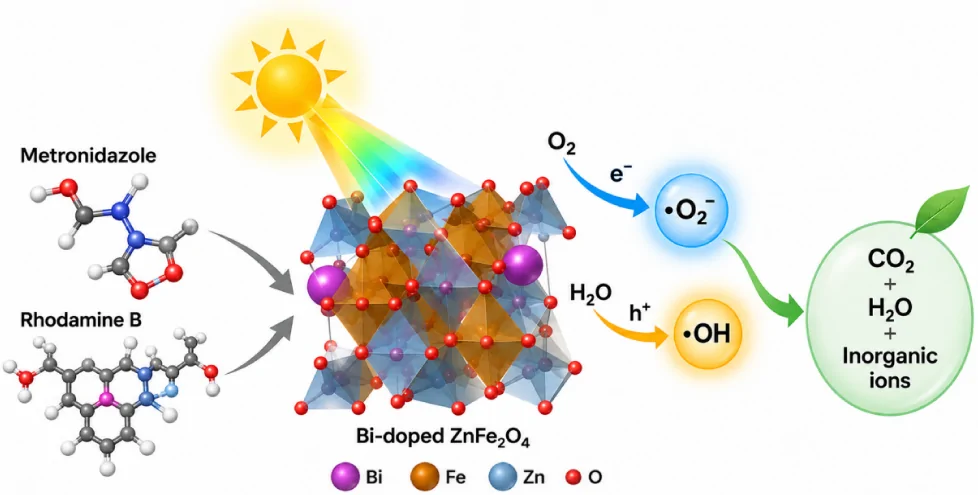

## 1 Introduction

The contamination of aquatic ecosystems by persistent organic pollutants, including pharmaceutical compounds such as MDZ and synthetic dyes such as RhB, has become a critical environmental concern due to their high chemical stability, toxicity, and resistance to conventional wastewater treatment processes.^1–4^ Their continuous discharge from industrial and domestic sources leads to long-term accumulation in water bodies, necessitating the development of efficient and sustainable remediation technologies.

Among advanced oxidation processes, semiconductor photocatalysis is considered one of the most promising strategies for environmental purification owing to its ability to harness solar energy and mineralize organic pollutants under mild conditions.^5,6^ However, its practical efficiency is still limited by rapid electron–hole recombination, insufficient visible-light absorption, and poor charge transport in many semiconductor systems. Spinel ferrites, particularly ZnFe_2_O_4_, have attracted significant attention as visible-light-active photocatalysts due to their narrow bandgap, structural stability, and magnetic recoverability.^7,8^

Nevertheless, the photocatalytic performance of ZnFe_2_O_4_ remains limited by the rapid recombination of photogenerated electron–hole pairs and the low mobility of charge carriers, which reduces charge-transfer efficiency and hinders its full utilization under visible-light irradiation.^9,10^ Overcoming these limitations has motivated extensive efforts in electronic structure engineering through approaches such as heterojunction construction, defect engineering, and cation doping. Previous studies have demonstrated that engineering the electronic structure of ZnFe_2_O_4_ through heterojunction formation or elemental doping can significantly improve its photocatalytic performance. For example, coupling ZnFe_2_O_4_ with graphene or g-C_3_N_4_, as well as introducing Cu or Ag species, has been shown to promote charge separation and broaden light absorption, thereby enhancing photocatalytic activity.^11–14^

Among the various dopants explored, Bi^3+^ has attracted particular interest owing to its unique electronic configuration and its ability to simultaneously modify the crystal structure and electronic properties of the host material. Owing to its 6s^2^ lone-pair electrons and larger ionic radius, Bi incorporation can induce local lattice distortions, promote oxygen-vacancy formation, and modify the electronic band structure.^15^ These effects collectively improve charge mobility, increase carrier lifetime, and create additional active sites for photocatalytic reactions. Indeed, enhanced charge-transport properties and superior photocatalytic performance have been reported for Bi-modified ZnFe_2_O_4_ systems, with the improvements attributed to oxygen-vacancy generation and more efficient charge separation.^16^

While these advances demonstrate the benefits of Bi incorporation, most reported systems are based on powders or nanoparticles, which present challenges related to catalyst recovery, aggregation, and long-term operation in continuous water-treatment processes. Transitioning from particulate catalysts to thin-film architectures offers a promising route to address these limitations while providing additional opportunities to tailor structural and electronic properties. In this regard, spray pyrolysis was selected as the fabrication method because it combines simplicity, low cost, scalability, and excellent control over film composition and uniformity, enabling the deposition of adherent large-area oxide coatings.^17–20^

Building on the synergistic advantages of Bi-induced electronic structure modulation and thin-film engineering, this work explores Bi-doped ZnFe_2_O_4_ thin films prepared by spray pyrolysis. The objective is to bridge the gap between dopant-enhanced ZnFe_2_O_4_ photocatalysts and practical thin-film photocatalytic systems by investigating their structural, optical, and photocatalytic behavior toward the degradation of RhB and MDZ under visible-light irradiation.

## 2 Experimental Section

### 2.1 Preparation of Pristine and Bi-ZnFe_2_O_4_ Thin Films

Pristine and bismuth-doped zinc ferrite (Bi-doped ZnFe_2_O_4_) thin films were synthesized using the spray pyrolysis method, owing to its versatility, cost-efficiency, and suitability for large-area deposition (the schematic of the main synthesis procedure is shown in Figure 1a. Glass substrates were first cleaned via sequential ultrasonication in acetone and ethanol (each for 10 min), followed by rinsing with high-purity deionized water and subsequent drying in a muffle furnace at 120 °C to ensure optimal surface cleanliness and wettability.

**Figure 1:**
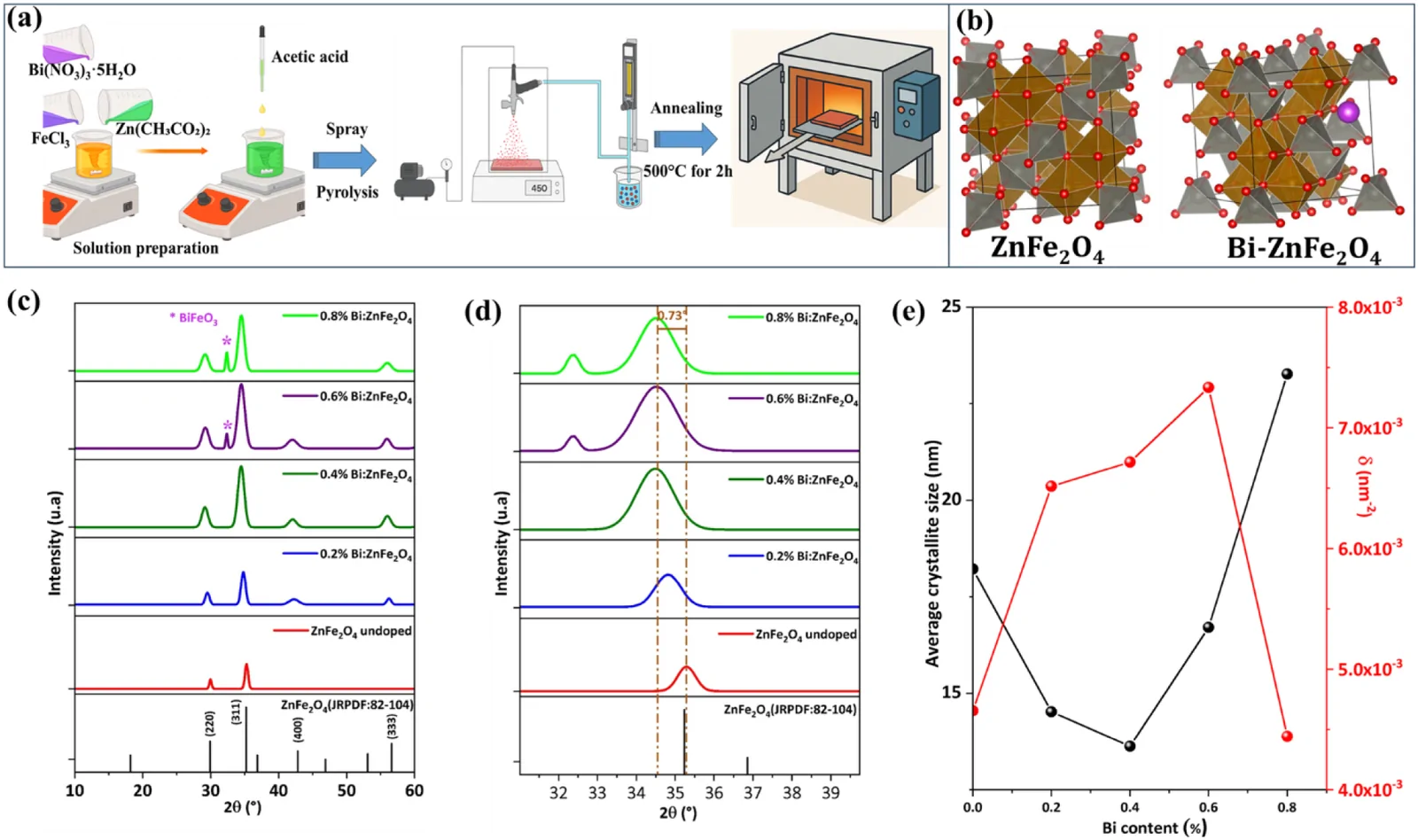
(a) synthesis process of Pristine and Bi-ZnFe_2_O_4_ thin films (b) Crystal structures of ZnFe_2_O_4_ and Bi-ZnFe_2_O_4_ (c) XRD patterns of pure and Bi-doped ZnFe_2_O_4_ samples with different Bi molar ratios (0.2–0.8%), (d) (311) peak shift with Bi level and (e) average crystallite size (D) and dislocation density (δ) as a function of Bi content.

The precursor solution for pristine ZnFe_2_O_4_ films was prepared by dissolving zinc acetate dehydrate (Zn­(CH_3_COO)_2_·2H_2_O, 99.99%) and iron­(III) chloride hexahydrate (FeCl_3_·6H_2_O, 98%) in a molar ratio of 1:2 (Zn^2+^:Fe^3+^), using a mixed solvent system of propanol and deionized water in a 3:1 volume ratio. To improve sol stability and suppress premature precipitation, 0.1 vol % of glacial acetic acid was added as a stabilizing agent. For the Bi-doped series, bismuth­(III) nitrate pentahydrate (Bi­(NO_3_)_3_·5H_2_O) was incorporated into the solution, adjusting the Zn/Bi weight ratio from 0% (pristine) up to 0.8%, to study the influence of dopant concentration on structural, optical, morphological, and photocatalytic properties. The selected low doping range was chosen based on previous reports indicating that small dopant concentrations are sufficient to tune structural and functional properties while maintaining phase stability and avoiding secondary phase formation in spinel oxide systems.^21–23^ Thin film deposition was carried out using a spray pyrolysis setup, with the substrate temperature precisely maintained at 450 ± 5 °C using a hot plate equipped with a proportional-integral-derivative (PID) temperature controller. A fixed nozzle-to-substrate distance of 27 cm and a constant spray rate of 10 mL/min were employed to ensure consistent droplet impact and uniform film morphology. Compressed air served as the carrier gas for atomization. Following deposition, all samples were thermally annealed in static air at 500 °C for 2 h (heating ramp rate: 10 °C/min) to enhance phase crystallization and relieve internal stress within the films. The thickness of the optimal 0.4% Bi-doped ZnFe_2_O_4_ thin film was measured using a surface profilometer (in Supporting Information Figure S1) and was found to be 178 nm.

### 2.2 Structural and Functional Characterization

The structural properties of the pristine and Bi-ZnFe_2_O_4_ thin films were examined using X-ray diffraction (XRD, Bruker D8 Advance, Cu Kα radiation, λ = 1.5418 Å) to determine phase composition, crystallinity, and crystallite size. Scanning electron microscopy (SEM) was employed to analyze surface morphology and microstructural features. Elemental composition and dopant distribution were investigated through energy-dispersive X-ray spectroscopy and mapping (EDX), ensuring spatial mapping of Zn, Fe, O, and Bi elements within the film matrix. X-ray photoelectron spectroscopy (XPS, PHI VersaProbe III) was utilized to probe the surface chemical states and oxidation states of the constituent elements. High-resolution spectra of Zn 2p, Fe 2p, and Bi 4f were recorded to assess chemical bonding and potential shifts associated with doping. Fourier-transform infrared spectroscopy (FTIR, Bruker Tensor II) was used to identify vibrational modes associated with metal–oxygen bonding within the spinel framework. Optical properties were characterized using UV–Vis-NIR spectroscopy (PerkinElmer LAMBDA 950) in reflectance mode across the spectral range of 250–1500 nm. The absorption data were analyzed using Tauc plots to estimate optical bandgap energies and evaluate the influence of dopant concentration on electronic transitions.

### 2.3 Photocatalytic Degradation of MDZ and RhB

The photocatalytic performance of Bi-ZnFe_2_O_4_thin films for different Bi doping (0.2, 0.4, 0.6, and 0.8 at.%) was evaluated through the absorbance and degradation of two model pollutants, MDZ and RhB, under simulated solar irradiation (AM 1.5G, 100 mW/cm^2^) to simulate realistic environmental remediation conditions. Thin film samples were individually immersed in 50 mL aqueous solutions of MDZ (10 mg/L) and RhB (10 mg/L) and maintained in the dark for 30 min to establish adsorption–desorption equilibrium, ensuring that subsequent concentration changes were attributable solely to photocatalytic activity. Based on all these results, 0.4 at.% of Bi is selected for further analysis. After equilibration, the samples were exposed to a calibrated solar simulator to initiate photocatalytic degradation, and the process was monitored over time by measuring solution absorbance with UV–Vis spectrophotometry. The residual concentrations of MDZ and RhB were quantified using their characteristic absorption peaks at 319 and 554 nm, respectively. This approach enabled a clear separation between dark adsorption and light-driven photocatalysis, providing reliable evidence of the effectiveness of Bi-ZnFe_2_O_4_ thin films as visible-light-active photocatalysts for removing pharmaceutical and dye contaminants from wastewater.

## 3 Results and Discussion

### 3.1 Structural Analysis

The crystal structure before and after Bi doping is shown in Figure 1b. The XRD pattern depicted in Figure 1c of pristine and Bi-ZnFe_2_O_4_ thin films (0.2, 0.4, 0.6, and 0.8 at.% Bi content, confirming the formation of a well-crystallized cubic spinel structure (JCPDS No. 22–1012) across all compositions. Distinct diffraction peaks corresponding to the (220), (311), and (422) planes are observed at 2θ ≈ 30.11°, 35.23°, and 55.6°, respectively. Among these, the (311) reflection at 35.23° is the most intense and dominant, signifying a strong preferred orientation along the (311) crystallographic plane. This preferential growth direction is characteristic of spinel ferrite thin films and suggests favorable energetic alignment and grain orientation during film formation. At higher Bi concentrations (0.6% and 0.8%), an additional peak appears at 2θ ≈ 32.4°, corresponding to the (110) plane of rhombohedral BiFeO_3_ (JCPDS No. 20-0169). The appearance of this diffraction peak indicates the formation of a BiFeO_3_ secondary phase, suggesting that the solubility limit of Bi in the ZnFe_2_O_4_ spinel lattice has been exceeded. This confirms that excess Bi is not fully incorporated into the ZnFe_2_O_4_ structure and instead segregates to form a separate BiFeO_3_ phase. Therefore, at higher doping levels, the system is better described as a mixed-phase ZnFe_2_O_4_/ BiFeO_3_ composite rather than a single-phase doped spinel. As illustrated in Figure 1d, the enlarged (311) diffraction peak demonstrates a pronounced shift of approximately 0.73° toward lower 2θ values with increasing Bi content, confirming significant lattice expansion within the ZnFe_2_O_4_ spinel structure. This pronounced shift results from the incorporation of larger Bi^3+^ ions (1.17 Å) into the Zn^2+^ sites, causing lattice expansion and increased microstrain. This observed peak shift confirms successful Bi incorporation at lower doping levels and directly reflects lattice expansion caused by the ionic size mismatch.

Table 1 and Figure 1e show that introducing small amounts of Bi (0.2–0.4%) into ZnFe_2_O_4_ decreases the average crystallite size from 18.2 to 13.6 nm and increases the dislocation density δ from 0.0046 to 0.0067 nm^–2^. This indicates that low Bi doping introduces lattice defects and distorts the spinel structure. These defects act as charge trapping sites and help suppress electron–hole recombination, which generally improves photocatalytic photodegradation efficiency. The enhanced photocatalytic performance at low Bi concentrations can also be associated with the reduction in crystallite size, which increases the effective surface area and provides a greater number of active sites for photocatalytic reactions. Similar correlations between smaller crystallite size, increased surface activity, and improved photocatalytic degradation efficiency have been reported for CuO and codoped ZnS thin films.^24,25^ Therefore, the combined effects of crystallite size reduction and defect generation are expected to facilitate charge carrier utilization and improve photodegradation performance. When the Bi content exceeds 0.4%, the crystallite size increases again (to 16.7 nm at 0.6% and 23.3 nm at 0.8%), and δ decreases. This change is attributed to the appearance of BiFeO_3_ peaks in the XRD patterns for the 0.6% and 0.8% samples. The formation of this secondary BiFeO_3_ phase means that excess Bi no longer enters the ZnFe_2_O_4_ lattice; instead, it segregates and promotes grain growth. As a result, the ZnFe_2_O_4_ crystallites become larger and contain fewer defects. This structural change has a direct impact on photodegradation. Larger crystallite size and lower defect density reduce the number of active sites and weaken charge trapping, which limits the formation of reactive species during photocatalysis. Therefore, samples with high Bi content (0.6–0.8%) are expected to show lower photodegradation activity.

**Table 1:** Structural Parameters of Undoped and Bi-Doped ZnFe_2_O_4_ Thin Films, Including Average Crystallite Size (D) and Microstrain (δ), Calculated for Various Bi Doping Contents

Sample	Average crystallite size (nm)	Dislocation density δ (nm^–2^)
Undoped ZnFe_2_O_4_	18.2	0.0046
0.2% Bi-ZnFe_2_O_4_	14.5	0.0065
0.4% Bi- ZnFe_2_O_4_	13.6	0.0067
0.6% Bi-ZnFe_2_O_4_	16.7	0.0073
0.8% Bi-ZnFe_2_O_4_	23.3	0.0044

### 3.2 Surface Morphology and Elemental Analysis

SEM analysis, as depicted in Figure 2a, reveals a pronounced morphological evolution induced by Bi doping in ZnFe_2_O_4_ films. The undoped ZnFe_2_O_4_ sample exhibits irregularly shaped and highly agglomerated grains with a wide size distribution, indicating uncontrolled grain growth and nonuniform nucleation. As depicted in Figure 2b, the EDS spectrum and elemental maps of the undoped film show the presence of O, Zn, and Fe as the main constituent elements. Elemental mapping reveals (Figure 2c–d) a uniform spatial distribution of O, Zn, and Fe throughout the film area, indicating good compositional homogeneity and the absence of secondary phase formation.

**Figure 2:**
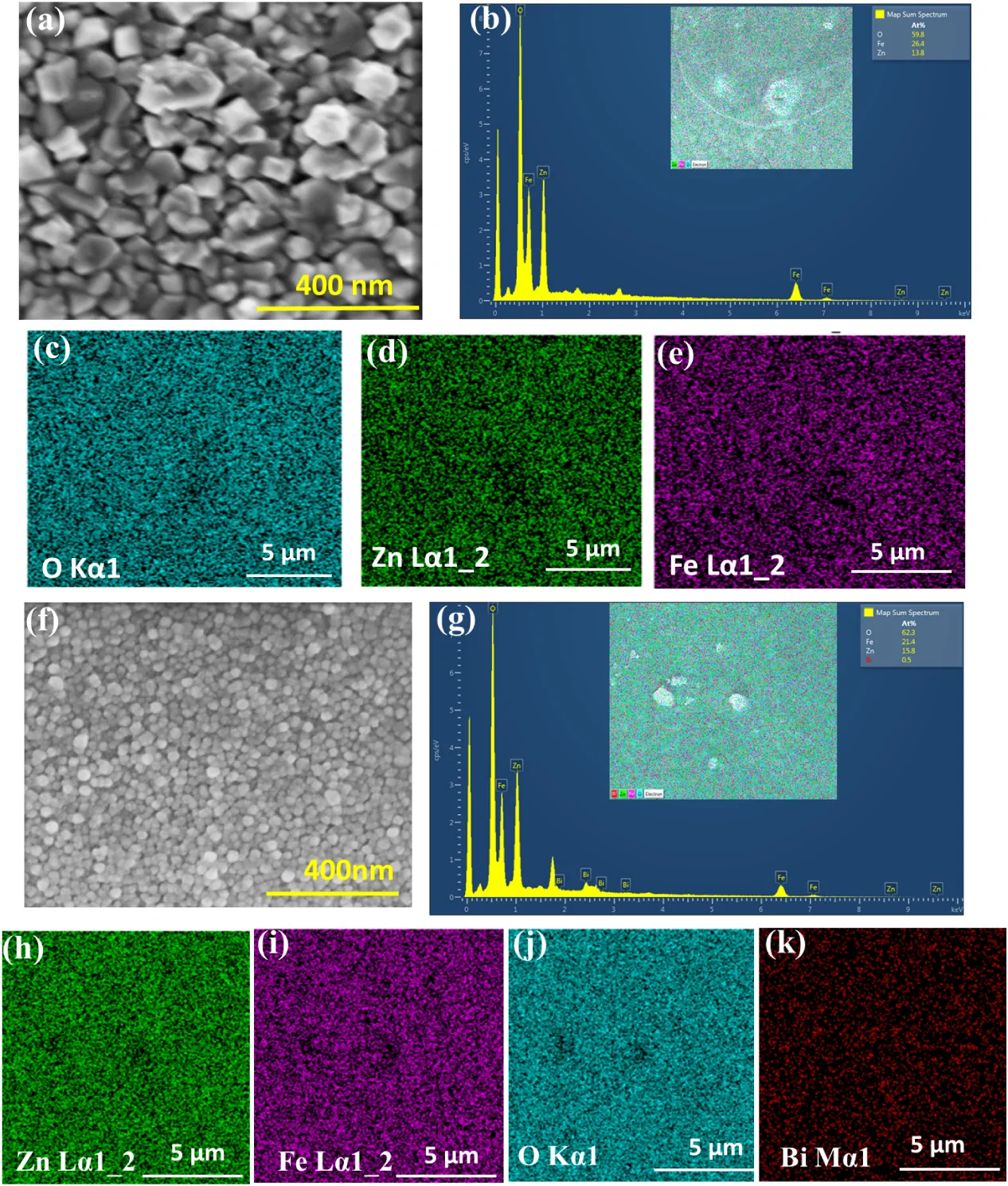
(a). SEM images, EDX spectra, and elemental maps of (b–e) undoped ZnFe_2_O_4_ and (f). SEM images and (g–k) 0.4% Bi: ZnFe_2_O_4_ thin films, respectively.

In contrast, the SEM image shown in Figure 2f for the Bi-doped ZnFe_2_O_4_ film reveals a more uniform and densely packed microstructure composed of finer, nearly spherical grains with reduced grain size compared to the undoped sample. This pronounced grain refinement suggests that Bi incorporation effectively suppresses grain growth and promotes homogeneous nucleation by modifying the crystallization kinetics during film formation. As a result, the reduction in grain size is expected to increase the exposed surface area available for interfacial reactions by providing a larger number of grain boundaries and surface-active sites. Additional SEM images at different magnifications are provided in Figure S2 (Supporting Information). Figure 2g EDS spectrum and elemental maps of the Bi-doped film confirming the presence of Zn, Fe, O, and Bi. The appearance of Bi peaks verifies successful Bi incorporation. Elemental maps shown in Figure 2h–k demonstrate a uniform distribution of all elements across the film surface, indicating homogeneous Bi doping and no observable phase segregation. This compositional uniformity, combined with the improved morphology, is expected to enhance charge transfer efficiency and provide many active sites, thereby contributing to the improved photocatalytic performance of Bi-doped ZnFe_2_O_4_.

### 3.3 FTIR Analysis

The FTIR spectra of undoped and Bi-doped ZnFe_2_O_4_ thin films (Figure 3a exhibit characteristic vibrations of the spinel lattice, with metal–oxygen stretching modes at 547 and 407 cm^–1^ corresponding to tetrahedral (A-site) and octahedral (B-site) cation–oxygen bonds.^26^ Broad bands at 3418 and 1609 cm^–1^ are assigned to O–H group stretching and O–H bending of adsorbed water,.^27,28^ The weak peak observed at 1381 cm^–1^ arises from C–O stretching of surface carbonates. Notably, the intensity of the M–O bands increase with Bi doping up to 0.4–0.6%, indicating enhanced local ordering and cation redistribution, before decreasing at 0.8% due to dopant-induced lattice disorder. These observations indicate that low-level Bi doping adjusts the spinel lattice, enhancing local vibrational order and cation arrangement without disrupting the overall structure.

**Figure 3:**
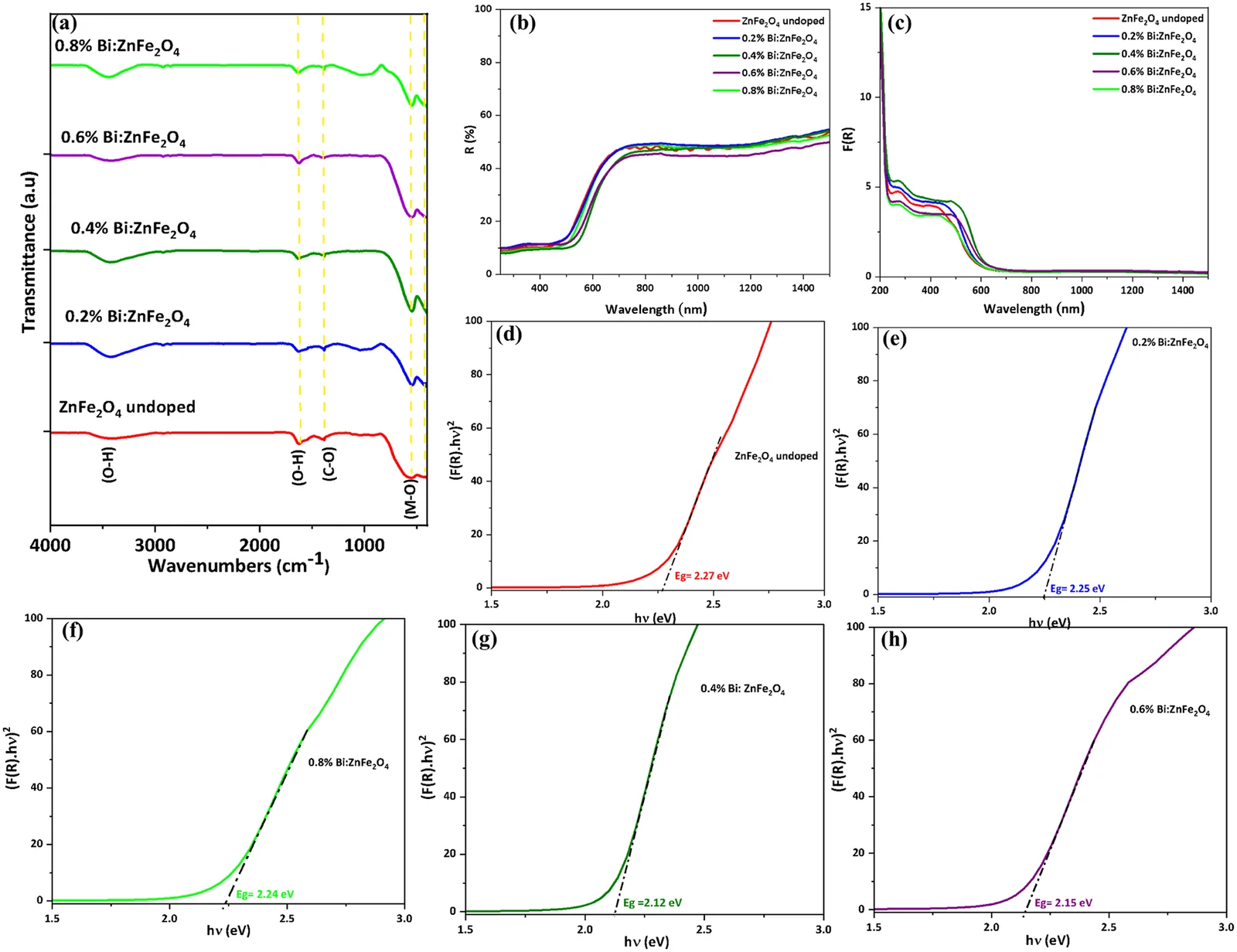
(a) FTIR spectra, (b) Reflectance spectra, (c) Kubelka–Munk functions, and (d–h) Tauc plots of pristine and Bi-doped ZnFe_2_O_4_ thin films (0.2–0.8 at.%).

### 3.4 Optical Analysis

The optical analysis of Bi-doped ZnFe_2_O_4_ thin films was investigated using UV–Vis reflectance spectroscopy. All samples show low reflectance in the UV–visible region (<600 nm) and higher reflectance in the NIR (>800 nm), as shown in Figure 3b, indicating strong visible-light absorption typical of ZnFe_2_O_4_. The 0.4% Bi-doped sample shows the highest Kubelka–Munk values in Figure 3c, indicating enhanced absorption, while higher doping levels (0.6–0.8%) slightly reduce absorption, likely due to structural distortion or minor BiFeO_3_ formation. The optical band gap (Eg) was determined from Tauc plots (Figure 3d–h), Table S1, Supporting Information, considering ZnFe_2_O_4_ as a direct allowed semiconductor. This assumption is also consistent with previous reports on ZnFe_2_O_4_ thin films prepared by spray pyrolysis and other deposition techniques. Salman et al. reported direct electronic transitions in ZnFe_2_O_4_ films synthesized by chemical spray pyrolysis based on their high absorption coefficient values (>10^4^ cm^–1^) at high photon energies.^30^ Furthermore, direct allowed transitions have been widely used for ZnFe_2_O_4_ thin films, where the optical band gap was determined using the relation (αhν)^2^= A­(hν-Eg).^31–33^ While bulk ZnFe_2_O_4_ has sometimes been reported as an indirect semiconductor, several studies on nanostructured ZnFe_2_O_4_ report direct band gap behavior instead. This shift from indirect to direct character is generally attributed to three factors. First, quantum confinement in small particles relaxes the selection rules and favors direct-type transitions; for example, direct band gap values of 2.11 and 2.30 eV have been reported for ZnFe_2_O_4_ nanoparticles and ZnFe_2_O_4_–TiO_2_ nanocomposites, respectively, both higher than the bulk value of 1.9 eV.^29^ Second, Nair et al. showed that increasing lattice strain in very small particles raises the surface pressure, which also increases the direct optical band gap.^30^ Third, defects and partial cation disorder, such as those introduced by doping, can further promote direct-type transitions near the band edge. The band gap values obtained in this work fall within the typical range reported for ferrite nanomaterials (1.9–2.5 eV),^31^ supporting the use of the direct transition model for our Bi-doped ZnFe_2_O_4_ thin films. We acknowledge, however, that the direct/indirect nature of ZnFe_2_O_4_ is still debated, and may depend on particle size, strain, and defect structure. The undoped film has Eg = 2.27 eV. Bi incorporation decreases Eg to 2.12 eV at 0.4 at.% due to electronic structure modifications induced by Bi^3+^ presence, including valence band restructuring and the introduction of intermediate energy states, as evidenced in XPS spectra below. At higher Bi concentrations (0.6–0.8 at.%), Eg increases, likely due to the formation of secondary BiFeO_3_ phases reducing defect-related absorption. This behavior is well correlated with XRD analysis, which shows that the 0.4 at.% Bi sample exhibits optimal crystallinity without secondary phases. The extracted Eg values for all compositions are summarized in Table S2 (Supporting Information), demonstrating the variation of the band gap with Bi content.

### 3.5 Surface and Electronic Structure Analysis

To analyze the surface composition, the XPS spectra of undoped and 0.4% Bi- ZnFe_2_O_4_. As shown in Figure S3 (Supporting Information), the XPS survey spectrum of pristine ZnFe_2_O_4_ confirms the presence of Zn, Fe, and O, whereas the Bi-doped ZnFe_2_O_4_ photocatalyst additionally exhibits Bi, confirming the successful incorporation of Bi into the ZnFe_2_O_4_ lattice. In the Zn 2p spectra (Figure 4b), the undoped sample exhibits a Zn 2p_3/2_ peak at 1020.99 eV; the position of the peak points toward the presence of Zn^2+^, while the doped sample shows a peak at 1021.32 eV, with a shift of 0.33 eV indicating that Bi incorporation changes the electronic environment around Zn. The Fe 2p XPS spectra (Figure 4a) reveal distinct electronic structures for undoped ZnFe_2_O_4_ and Bi: ZnFe_2_O_4_. In undoped ZnFe_2_O_4_, the Fe 2p3/2 and Fe 2p1/2 peaks appear at 710.73 and 724.60 eV, respectively, accompanied by characteristic satellite features at 715.73 and 733.27 eV, confirming the presence of Fe^3+^ ions in octahedral coordination within the spinel lattice. Upon Bi doping, the main Fe 2p peaks slightly shift to 710.60 and 724.87 eV, and additional components emerge at 712.48 and 719.07 eV, indicating the coexistence of Fe^3+^ and Fe^2+^ states. This partial reduction of Fe^3+^ to Fe^2+^ serves as a charge-compensation mechanism induced by Bi^3+^ incorporation while preserving the spinel framework. To preserve charge neutrality, a fraction of Fe^3+^ ions at the octahedral sites undergo reduction to Fe^2+^. This charge-compensation mechanism is consistent with previous reports on Cr^3+–^doped ferrites, where substitution by trivalent dopants induces partial Fe^3+^ → Fe^2+^ reduction in the octahedral sublattice.^32^ The resulting mixed-valence electronic structure enhances charge-carrier mobility and electron-transfer processes, contributing to reduced recombination and improved visible-light-driven photocatalytic performance compared to undoped ZnFe_2_O_4_. O 1s spectra (Figure 4c) present three components: lattice oxygen at ∼529.8 eV, defect-related oxygen at ∼531.3–531.5 eV, and adsorbed oxygen species at ∼532.3–532.5 eV, with slight upshifts in the doped sample indicating modified oxygen environments. The Bi 4f spectrum depicted in Figure 4d shows peaks at 158.96 and 164.27 eV, corresponding to Bi 4f_7/2_ and Bi 4f_5/2_ levels, confirming the presence of Bi^3+^. The XPS analysis confirms Zn^2+^, Fe^3+^, and O^2–^ as the dominant species, with Bi^3+^ doping introducing partial Fe^3+^ reduction and subtle modifications in the Zn–O and O environments while preserving the spinel structure.

**Figure 4:**
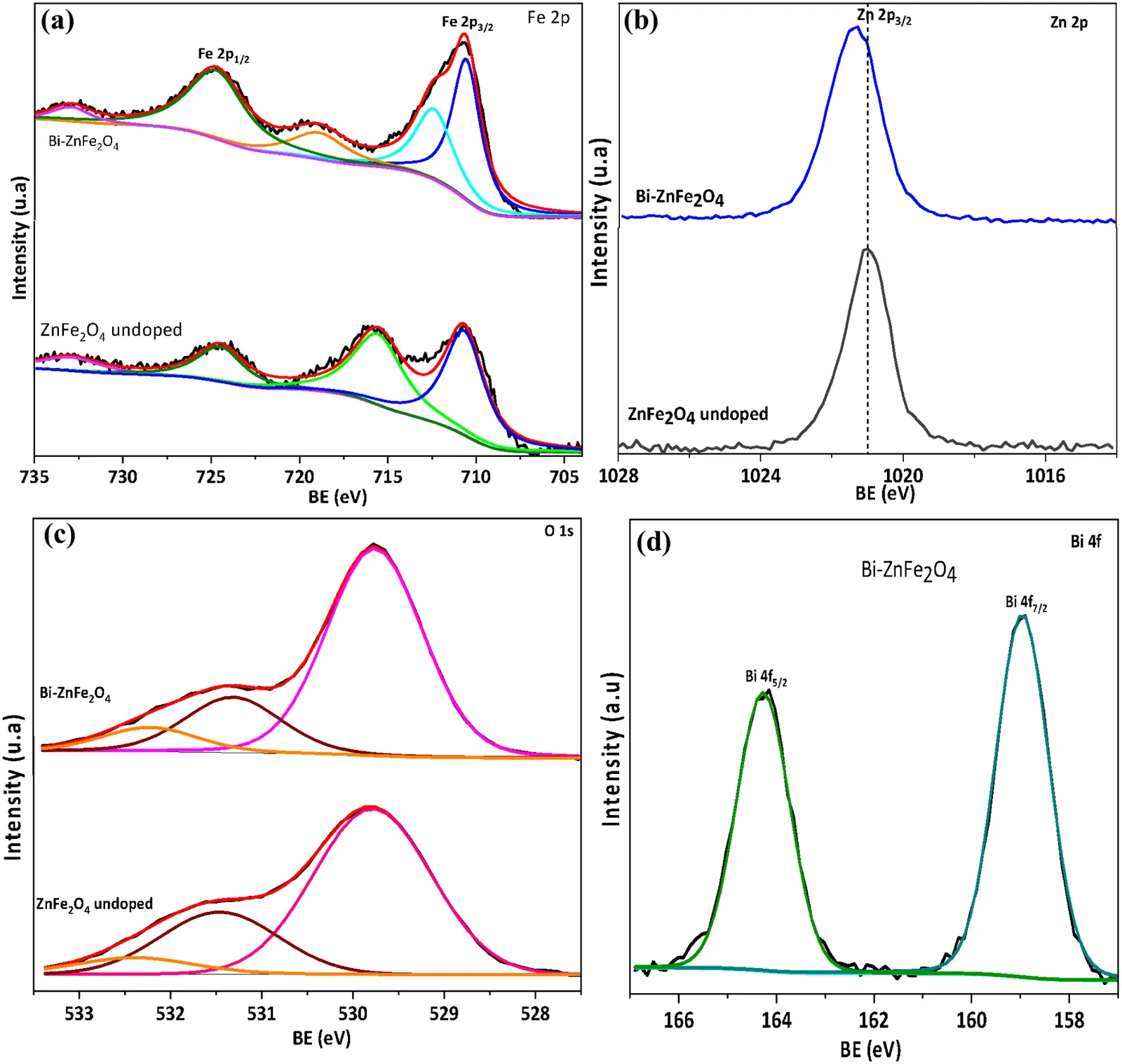
XPS spectra of pure and 0.4% Bi-doped ZnFe_2_O_4_ samples of (a) Fe 2p, (b) Zn 2p, (c) O 1s, and (d) Bi 4f.

Although XPS confirms the presence of Bi^3+^ and the modification of the local chemical environment, it cannot independently determine the exact crystallographic site occupied by Bi^3+^ ions. Therefore, the possible incorporation mechanism was discussed based on previous reports on Bi-doped spinel ferrites. In related work Mn_0.6_Zn_0.4_Bi_*x*_Fe_2–x_O_4_ ferrites, Bi^3+^ ions were reported to preferentially occupy octahedral sites, inducing lattice expansion and cation redistribution between tetrahedral and octahedral positions.^33^ Similarly, Mössbauer spectroscopy confirmed the incorporation of Bi^3+^ through partial substitution of Fe^3+^ ions in CoBi_*x*_Fe_2–x_O_4_ ferrites, accompanied by modifications in cation distribution and magnetic interactions.^34^ Furthermore, Bi substitution at Fe-related sites was suggested in Ni_0.5_Zn_0.5_Bi_*x*_Fe_2–x_O_4_ ferrites based on structural and vibrational analyses,^35^ while FTIR studies of Bi-doped zinc ferrites revealed changes in octahedral metal–oxygen vibrations associated with Bi incorporation.^36^ Thus, based on the present XPS results and previous literature, Bi^3+^ incorporation into Fe-related sites of the ZnFe_2_O_4_ lattice can be considered a plausible mechanism; however, its exact occupation site requires further confirmation using advanced techniques.

Collectively, these electronic structure modifications provide direct mechanistic justification for the optical band gap narrowing from 2.27 to 2.12 eV reported in Figure 3d–h. We can understand three concurrent mechanisms from the XPS data. (i) the confirmed Bi^3+^ species, evidenced by Bi 4f peaks at 158.96 and 164.27 eV, possesses lone pair electrons that hybridize with O 2p orbitals at the valence band maximum, raising the valence band edge and directly narrowing the band gap; (ii) the partial Fe^3+^ → Fe^2+^ reduction, confirmed by the emergence of new Fe 2p components at 712.48 and 719.07 eV, generates intermediate energy states within the band gap that reduce the optical transition energy and contribute to the observed red shift in light absorption; and (iii) the positive binding energy shifts recorded in both the Zn 2p (+0.33 eV) and O 1s spectra reflect enhanced Bi–O bond covalency relative to Zn–O bonds, broadening the valence band through greater orbital overlap and further contributing to band gap narrowing. These three XPS-evidenced mechanisms acting simultaneously account for the measured band gap reduction and the consequent enhancement in visible-light harvesting, establishing a direct and complete structure–property correlation in Bi-doped ZnFe_2_O_4_.

#### 3.5.1 Photodegradation of RhB

The photocatalytic activity of the prepared thin films, both pristine and Bi-doped, was evaluated using the degradation of RhB under solar simulator irradiation. The absorbance spectra presented in Figure 5a–c illustrate the progressive decrease in RhB concentration with increasing irradiation time for the undoped ZnFe_2_O_4_, 0.4% Bi: ZnFe_2_O_4_and 0.6% Bi-ZnFe_2_O_4_ samples, respectively. The clear reduction in the main absorption peak intensity indicates that Bi incorporation significantly enhances the photocatalytic response compared to the undoped film. To further quantify the degradation process, the temporal variation of RhB degradation efficiency is shown in Figure 5e, while the corresponding kinetic analysis is presented in Figure 5d. All samples followed pseudo-first-order kinetics, but with different rate constants depending on the Bi content. The pristine ZnFe_2_O_4_ film showed the lowest photocatalytic performance, with a slower rate of RhB decomposition. In contrast, both Bi-doped films exhibited improved degradation rates, confirming that Bi incorporation promotes charge separation and reduces recombination of photogenerated carriers.

**Figure 5:**
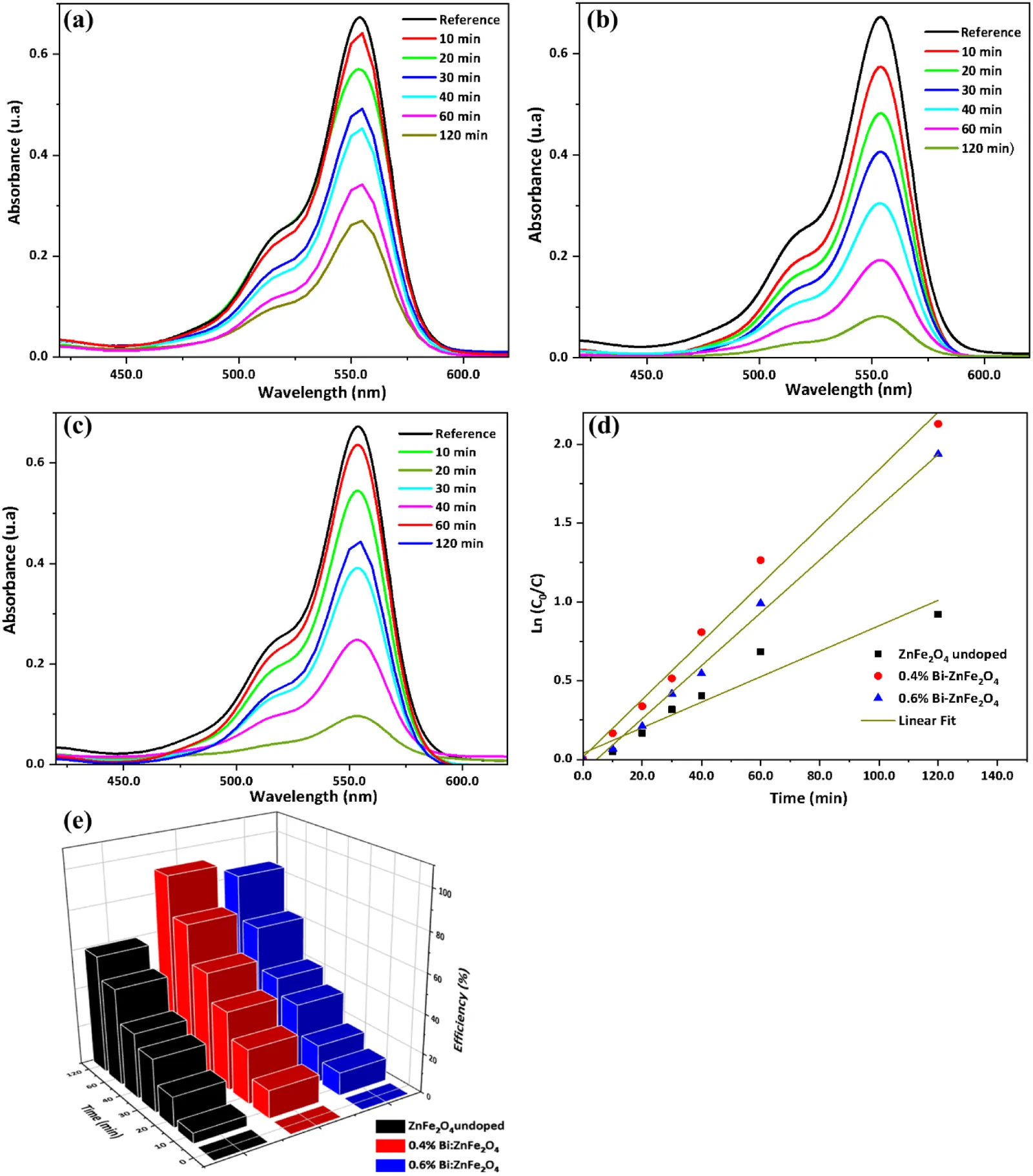
UV–Vis absorbance spectra of RhB during photodegradation using (a) undoped ZnFe_2_O_4_, (b) 0.4% Bi- ZnFe_2_O_4_, and (c) 0.6% Bi- ZnFe_2_O_4_ thin films. (d) Kinetics curve of the pseudo-first-order equation and (e) Photodegradation efficiency of RhB under visible-light irradiation for undoped and Bi- ZnFe_2_O_4_thin films.

Notably, the 0.4% Bi-ZnFe_2_O_4_ thin film exhibited the highest catalytic activity, achieving an efficiency of 88.9% after 120 min of irradiation. The 0.6% Bi-ZnFe_2_O_4_ sample also showed enhanced activity compared to the undoped film, achieving 85.1% efficiency, while the undoped ZnFe_2_O_4_ thin film reached only 60.2% degradation under the same conditions. The slightly lower performance of the 0.6% Bi-doped sample compared to the 0.4% counterpart is directly correlated with the appearance of a BiFeO_3_ secondary phase, as confirmed by XRD analysis. Among the secondary-phase-containing compositions (0.6% and 0.8%), the 0.6% sample was selected for photocatalytic evaluation because it exhibits more favorable structural and optical properties, namely a smaller crystallite size, which offers greater active surface area, and a lower band gap, enabling broader visible-light absorption compared to the 0.8% sample. Nevertheless, despite these relative structural advantages, the 0.6% sample still underperforms the phase-pure 0.4% Bi-ZnFe_2_O_4_, demonstrating that BiFeO_3_ formation introduces recombination centers at heterogeneous interfaces that measurably suppress charge separation and photocatalytic efficiency, regardless of other favorable structural parameters. These results demonstrate that moderate Bi doping significantly improves the photocatalytic performance of ZnFe_2_O_4_ thin films, with the optimum performance observed for the 0.4% Bi-doped sample. The higher efficiency highlights the critical role of controlled Bi incorporation in enhancing charge transfer dynamics and broadening the visible-light response, which makes ZnFe_2_O_4_ Bi-ZnFe_2_O_4_ a promising photocatalyst for organic dye degradation. To analyze the RhB photocatalytic performance of the Bi-doped and undoped ZnFe_2_O_4_ thin films, the degradation kinetics were evaluated using the Langmuir–Hinshelwood (L–H) model, which is widely employed to describe heterogeneous photocatalytic reactions. Based on the pseudo-first-order approximation, the degradation rate is proportional to the pollutant concentration, and the apparent rate constant (*k*_1_) can be extracted from the linear fit of the kinetic plot. The relationship is expressed as the equation below:

1 $$Ln\left(\frac{C_{0}}{C}\right)=k_{1}.t$$

where *k*_1_ is the pseudo-first-order rate constant, and C_0_ and C correspond to the initial and final dye concentrations, respectively. As shown in Figure 5d, all thin films exhibit clear pseudo-first-order kinetics, confirming that the degradation process follows the L–H model.

The kinetic analysis of RhB degradation (Table 2) demonstrates that Bi incorporation significantly enhances the photocatalytic activity of ZnFe_2_O_4_ thin films. The undoped ZnFe_2_O_4_ sample exhibits the lowest apparent rate constant (*k* = 0.00809 ± 0.00094 min^–1^), indicating a slower degradation process. After introducing 0.4% Bi, the rate constant increases to 0.01824 ± 0.00089 min^–1^, corresponding to more than a 2-fold enhancement in the degradation rate. This improvement can be attributed to the beneficial role of Bi incorporation in modifying the electronic structure of ZnFe_2_O_4_, promoting visible-light absorption and facilitating the separation of photogenerated charge carriers. Although the 0.6% Bi-doped sample also exhibits improved photocatalytic performance (*k* = 0.01678 ± 0.00055 min^–1^), its slightly lower rate constant compared with the 0.4% Bi sample suggests that excessive Bi incorporation may lead to defect formation or act as recombination centers, reducing the efficiency of charge utilization.

**Table 2:** Values of Rate Constants (*k*_1_), Fitting Errors and R^2^ Values for Different Studied Samples

Sample	Rate constant (min^–1^)	Fitting error (±) (min^–1^)	Coefficient of determination R^2^
Undoped ZnFe_2_O_4_	0.00809	0.000942	0.93646
0.4% Bi-ZnFe_2_O_4_	0.01824	0.000889	0.98825
0.6% Bi-ZnFe_2_O_4_	0.01678	0.000552	0.99461

The reliability of the kinetic fitting was evaluated by considering both the coefficient of determination and the fitting uncertainty. The obtained R^2^ values (0.93646–0.99461) together with the relatively low fitting errors confirm the suitability of the pseudo-first-order model for describing RhB degradation under the investigated conditions. According to the Langmuir–Hinshelwood mechanism, the pseudo-first-order approximation is applicable when the pollutant concentration is sufficiently low, and the adsorption equilibrium between pollutant molecules and active sites remains nearly constant throughout the reaction. In this study, the low initial RhB concentration indicates that the surface coverage of the catalyst remains approximately stable during the degradation process. The linear fitting behavior observed over the entire irradiation period further indicates that the adsorption equilibrium can be considered approximately constant throughout the degradation process. Therefore, the pseudo-first-order model provides an appropriate description of the photocatalytic degradation kinetics, although minor deviations from ideal Langmuir–Hinshelwood behavior may occur at extended irradiation times due to the progressive decrease in RhB concentration and changes in surface adsorption conditions.

#### 3.5.2 Photodegradation of MDZ

Following the comprehensive photocatalytic screening conducted using RhB as a model pollutant, the study was extended to metronidazole (MDZ), a pharmaceutical contaminant of growing environmental concern. Unlike RhB, which is a dye molecule characterized by strong chromophoric absorption and high surface affinity, MDZ represents a structurally distinct and considerably more challenging target, exhibiting weaker visible-light absorption and a fundamentally different degradation pathway, making its inclusion essential for demonstrating the true photocatalytic versatility of the synthesized thin films. The RhB screening was deliberately structured to fulfill a dual scientific objective: to identify the optimal Bi doping concentration and to explicitly evaluate whether the BiFeO_3_ secondary phase appearing at 0.6% Bi doping confers any photocatalytic benefit. The answer was clear and decisive. The 0.4% Bi-doped ZnFe_2_O_4_ sample, the only phase-pure Bi-doped composition free of secondary-phase contamination, exhibited the highest degradation efficiency (89%). In contrast, the 0.6% Bi-doped sample, containing a BiFeO_3_ secondary phase, achieved a lower efficiency (85%), while the undoped ZnFe_2_O_4_ showed the poorest performance (60.2%). This result carries an important physical message: it is the selective incorporation of Bi into the ZnFe_2_O_4_ lattice, without structural disruption from secondary phase formation, that drives the enhancement in photocatalytic performance. The presence of BiFeO_3_ at 0.6% doping does not add value; it dilutes it. Based on the conclusive outcomes of the RhB screening, only two samples were selected for further investigation: the undoped ZnFe_2_O_4_ and the optimal 0.4% Bi-doped composition. These two samples are now evaluated against (MDZ), a structurally complex and pharmaceutically persistent pollutant that represents a fundamentally different and far more challenging degradation target than a model dye molecule.

The absorbance spectra of MDZ during irradiation are shown in Figure 6a and b for the undoped and 0.4% Bi-ZnFe_2_O_4_films, respectively. A pronounced reduction in MDZ absorbance intensity is observed with the Bi-doped film compared to the undoped counterpart, indicating much faster photocatalytic decomposition. The degradation efficiencies, summarized in Figure 6c, clearly highlight this improvement: after 120 min of visible-light irradiation, the undoped ZnFe_2_O_4_ thin film achieved only 30% MDZ removal, whereas the 0.4% Bi-ZnFe_2_O_4_ thin film reached a much higher efficiency of 75%. This remarkable enhancement can be attributed to the ability of Bi doping to (i) improve visible-light absorption, (ii) facilitate electron–hole separation, and (iii) promote the generation of reactive oxygen species (ROS), all of which are essential for the breakdown of pharmaceutical molecules such as MDZ. It is noteworthy that the improvement observed for MDZ degradation is even more pronounced than that for RhB. While RhB degradation efficiency increased from 60.2% (undoped) to 89% (0.4% Bi), MDZ degradation increased from only 31% (undoped) to 75% (0.4% Bi), representing almost a 4-fold relative enhancement. This demonstrates that Bi doping is particularly beneficial for more recalcitrant pollutants such as pharmaceuticals, where direct photolysis and adsorption are less effective. Taken together, the results from both RhB and MDZ establish 0.4% Bi-ZnFe_2_O_4_ as the optimal composition among the studied thin films, capable of efficiently degrading both dye-type and pharmaceutical-type contaminants under visible light. This dual validation strengthens the case for Bi-ZnFe_2_O_4_ thin films as promising photocatalysts for diverse classes of organic pollutants in water treatment applications.

**Figure 6:**
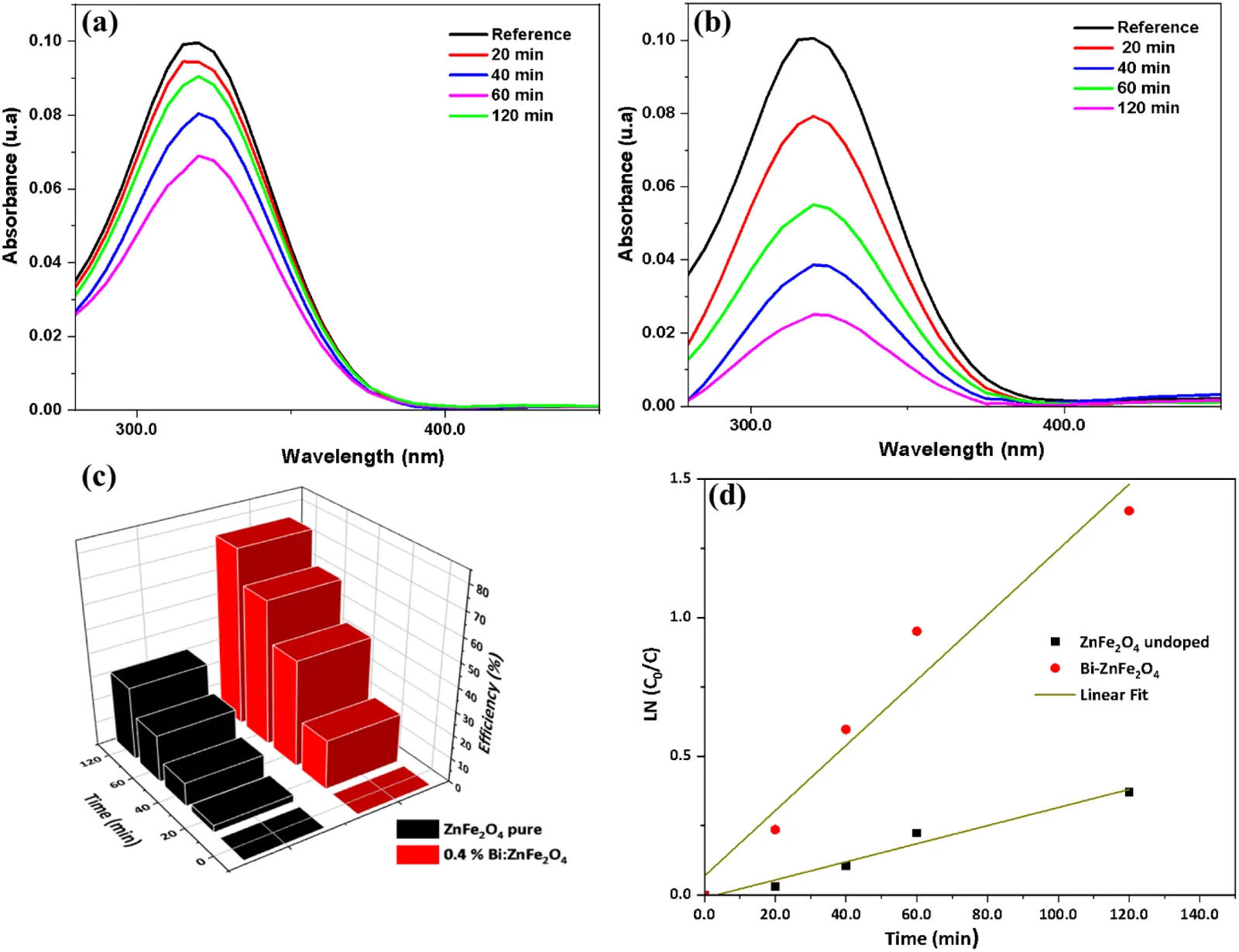
UV–Vis absorbance spectra of MDZ during photodegradation using (a) undoped ZnFe_2_O_4_, (b) 0.4% Bi-doped ZnFe_2_O_4_ thin films, (c) Photodegradation efficiency of MDZ under visible-light irradiation, and (d) Kinetics curve of the pseudo-first-order equation for undoped and 0.4% Bi-doped ZnFe_2_O_4_ thin films.

The photocatalytic kinetic (Table S2, Supporting Information) analysis confirms the positive effect of Bi incorporation on MDZ degradation. The undoped ZnFe_2_O_4_ film exhibits a rate constant of *k* = 0.00327 ± 0.00031 min^–1^, whereas the 0.4% Bi- ZnFe_2_O_4_ sample shows a more than 3-fold increase (*k* = 0.01178 ± 0.00144 min^–1^), indicating enhanced photocatalytic activity. This improvement can be attributed to Bi-induced modification of the electronic structure, leading to more efficient charge separation and increased reactive oxygen species generation. The satisfactory R^2^ values (0.97364 and 0.95678) and low fitting errors confirm the reliability of the pseudo-first-order kinetic model. Considering the low MDZ concentration, the Langmuir–Hinshelwood assumption of approximately constant surface adsorption during the degradation process remains valid.

The reusability and stability of the optimal 0.4% Bi-doped ZnFe_2_O_4_ thin film were evaluated through four consecutive photocatalytic degradation cycles of RhB under identical operating conditions. As illustrated in Figure 7a, the degradation efficiency decreased only slightly from 89% in the first cycle to 85% after the fourth cycle, demonstrating the excellent recyclability of the photocatalyst. The limited loss in activity (∼4%) may be attributed to the partial adsorption of reaction intermediates on the catalyst surface or minor changes in the surface-active sites during repeated operation. Nevertheless, the photocatalytic performance remained largely preserved, indicating that the Bi-doped ZnFe_2_O_4_ thin film possesses good operational stability.

**Figure 7:**
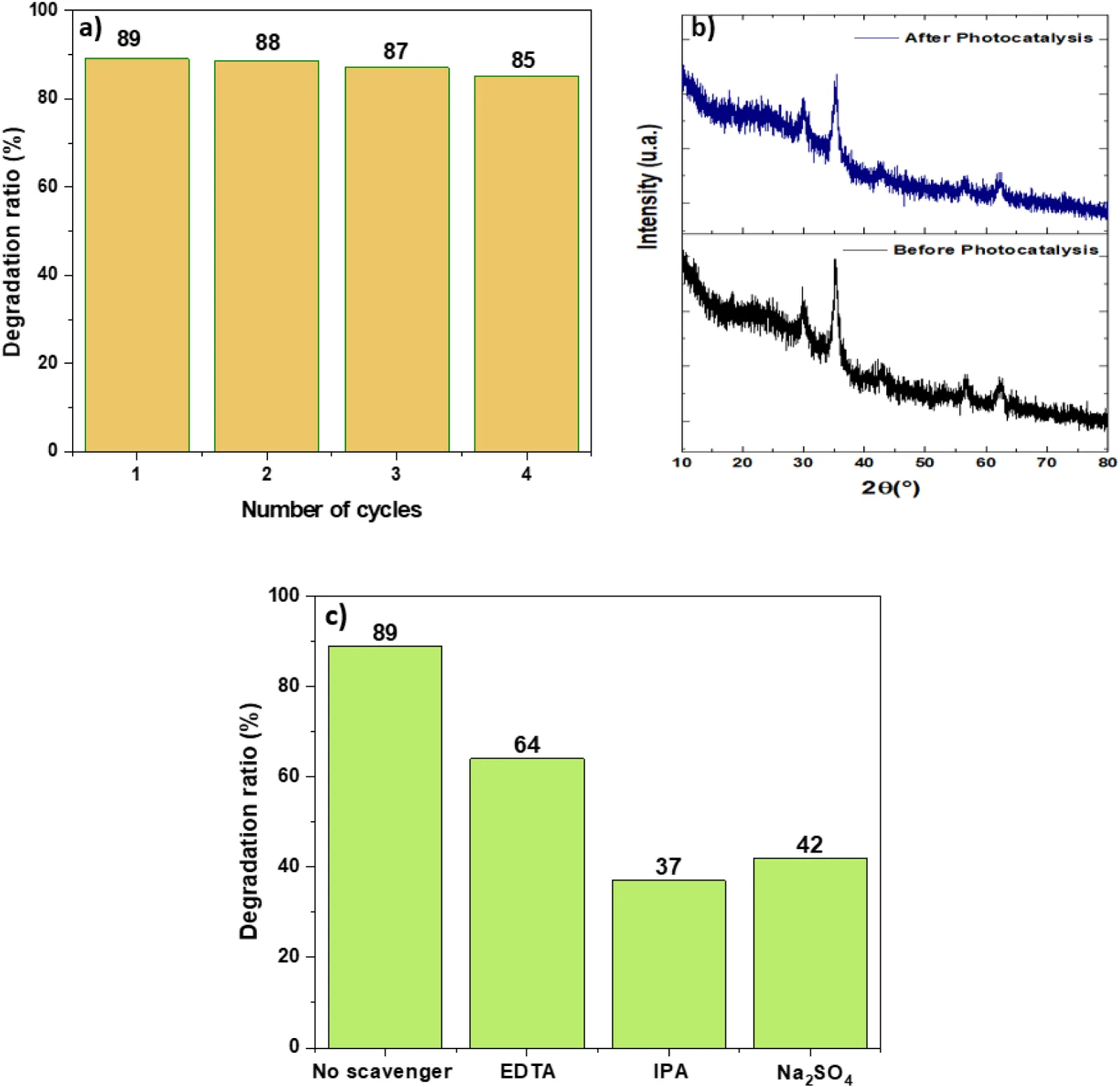
(a) Final degradation ratios of RhB over 0.4% Bi-doped ZnFe_2_O_4_ for 120 min in four cycles, (b) XRD patterns of the thin film before and after photocatalytic degradation, and (c) Photocatalytic Degradation of RhB over 0.4% Bi-Doped ZnFe_2_O_4_ under Visible Light Irradiation (120 min).

To further investigate the structural integrity of the photocatalyst after repeated use, XRD analyses were performed before and after the photocatalytic cycling tests (Figure 7b). The diffraction patterns exhibited the characteristic peaks of the spinel ZnFe_2_O_4_ phase with no noticeable peak shifts, disappearance of reflections, or emergence of secondary phases after photocatalysis. The preservation of the crystal structure confirms the high structural stability of the deposited thin film under irradiation and stirring conditions. Moreover, no visible film detachment was observed throughout the cycling experiments, indicating strong adhesion of the photocatalyst to the substrate. These findings demonstrate that the 0.4% Bi-doped ZnFe_2_O_4_ thin film maintains both its photocatalytic activity and structural integrity during repeated use, highlighting a significant practical advantage over powder photocatalysts, which typically require post-treatment separation and are more susceptible to material loss during recovery. It is worth noting that RhB was selected as a representative model pollutant for evaluating the recyclability and structural stability of the photocatalyst, as these properties are mainly related to the intrinsic durability of the catalyst rather than the nature of the pollutant molecule. The obtained results confirmed the excellent stability of the 0.4% Bi-doped ZnFe_2_O_4_ thin film during repeated photocatalytic operation. Further investigations involving long-term cycling tests and postreaction characterization during MDZ degradation would provide additional insights into the catalyst behavior under different degradation environments.

To identify the dominant reactive species involved in the photocatalytic degradation of RhB, scavenger trapping experiments were systematically performed under identical reaction conditions using specific quenching agents. The pristine system exhibited a degradation efficiency of 89%, confirming the strong photocatalytic activity of 0.4%Bi-doped ZnFe_2_O_4_. Upon addition of EDTA, the degradation efficiency decreased to 64%, indicating that photogenerated holes (h^+^) participate in the oxidation process, although they are not the primary active species. A more pronounced decrease was observed with isopropanol (IPA), where the efficiency dropped significantly to 37%, confirming that hydroxyl radicals (•OH) are the dominant reactive oxygen species responsible for RhB degradation. In contrast, the addition of Na_2_SO_4_ resulted in a reduction of the degradation efficiency to 42%. Since Na_2_SO_4_ is not a radical scavenger, this decrease is attributed to increased ionic strength and electrostatic shielding effects, which weaken the adsorption of RhB molecules on the catalyst surface and hinder interfacial charge transfer rather than directly quenching reactive species. Overall, these results clearly demonstrate that •OH radicals are the main oxidizing species, with photogenerated holes contributing to a lesser extent, while adsorption and surface charge interactions also play an important role in governing the overall photocatalytic performance.

#### 3.5.3 Photodegradation Mechanism of RhB and MDZ

The photodegradation process on Bi- doped ZnFe_2_O_4_ thin films starts with the absorption of visible-light photons by the semiconductor, which excites electrons from the valence band (VB) to the conduction band (CB), thereby generating electron–hole pairs (e^–^ – h^+^):

2 $${\mathrm{Bi:}\mathrm{ZnFe}}_{2}O_{4}+h\nu \to e_{CB}^{-}+h_{VB}^{+}$$

The key advantage of Bi doping lies in the introduction of quasi-stable energy levels within the band gap, which act as migration centers for the photogenerated charge carriers, effectively delaying their recombination and promoting their spatial separation. This enhanced charge separation facilitates the generation of reactive oxygen species (ROS) through the following interfacial redox reactions:

3 $$O_{2}+e_{CB}^{-}\to O_{2}^{\cdot -}$$

4 $$h_{VB}^{+}+H_{2}O\to \cdot \mathrm{OH}+H^{+}$$

5 $$h_{VB}^{+}+{\mathrm{OH}}^{-}\to \cdot \mathrm{OH}$$

The resulting superoxide anions $\left(O_{2}^{\cdot ‐}\right)$ and hydroxyl radicals (·OH) are responsible for the degradation of organic pollutants (*R*) into harmless end products such as CO_2_ and H_2_O:

6 $$R+\mathrm{ROS}\to {\mathrm{CO}}_{2}+H_{2}O+\mathrm{mineral residues}$$

The above mechanism, presented in (Supporting Information) Figure S4, governs the photocatalytic degradation of organic pollutants on Bi-doped ZnFe_2_O_4_ thin films. These reactive oxygen species (ROS), mainly superoxide $\left(O_{2}^{\cdot ‐}\right)$ and hydroxyl radicals (•OH), are highly reactive and attack the chemical bonds of both RhB and MDZ molecules. This leads to the gradual breakdown of their structures through bond cleavage and fragmentation, converting them into smaller, less complex intermediates, and finally into CO_2_, H_2_O, and inorganic ions through complete mineralization.

Recent studies have demonstrated that Bi^3+^ incorporation is an effective strategy for improving the visible-light photocatalytic performance of spinel ferrite materials. The introduction of Bi into ferrite structures can modify their electronic properties, reduce the band gap, enhance visible-light absorption, and promote more efficient charge carrier separation. Accordingly, several studies have explored Bi-doped spinel ferrites as promising photocatalysts, reporting enhanced photocatalytic activity and improved degradation efficiencies under visible-light irradiation compared with their undoped counterparts. El Bassami et al. reported that Bi-doped Mg–Ni ferrite (Mg_0.7_Ni_0.3_Bi_*x*_Fe_2–x_O_4_) exhibited excellent visible-light photocatalytic activity toward Rhodamine B degradation, which was attributed to the modified optical properties and enhanced stability of the Bi-containing ferrite system.^31^ Similarly, Bi^3–^-doped Ni–Cu–Cr quinary ferrite nanoparticles showed remarkable photocatalytic activity for Rhodamine B degradation, where the improved efficiency was associated with reduced band gap energy, increased active sites, and reactive oxygen species generation.^30^ Furthermore, Bi incorporation into Co_0.5_Ni_0.25_Zn_0.25_Fe_2–x_O_4_ ferrite enhanced methylene blue degradation under visible-light irradiation by improving light absorption and charge migration.^37^ Bi-doped Ni–Co ferrite nanoparticles also demonstrated enhanced photocatalytic activity toward Congo red removal, confirming the beneficial role of Bi substitution in tuning the surface and electronic properties of spinel ferrites.^38^ These reports collectively demonstrate the beneficial role of Bi modification in tuning the structural, optical, and electronic properties of spinel ferrites, thereby enhancing their visible-light-driven photocatalytic activity. Such improvements in light harvesting, charge carrier dynamics, and reactive species generation highlight the potential of Bi incorporation as an effective strategy for developing high-performance ferrite photocatalysts. Therefore, the photocatalytic performance of the Bi-doped ZnFe_2_O_4_ thin films developed in the present study was further assessed through comparison with other reported photocatalytic systems.

In this context, the photocatalytic efficiency of Bi-doped ZnFe_2_O_4_ thin films was compared with other photocatalysts reported in the literature (Table S3, Supporting Information). The present system achieved 89% degradation of RhB and 75% degradation of MDZ within 120 min of visible-light irradiation, a performance that compares favorably with several previously reported materials. Notably, comparable or even higher degradation efficiencies reported for other systems, such as Bi-doped ZnO^39^ and SBA-15/TiO_2_,^40^ were only achieved using UV irradiation and considerably longer treatment times (300 and 200 min, respectively), whereas the present system reaches similar or better performance under visible light within a shorter irradiation period. This highlights the practical advantage of Bi-doped ZnFe_2_O_4_ thin films, which combine fast, visible-light-driven degradation, a more energy-efficient and readily available excitation source than UV, with effective removal of both dye and pharmaceutical pollutants.

## 4 Conclusion

In this work, undoped and Bi-doped ZnFe_2_O_4_ thin films were successfully synthesized by spray pyrolysis and systematically studied to evaluate the effect of Bi incorporation on their structural, morphological, optical, electronic, and photocatalytic properties. XRD results confirmed the formation of a pure spinel phase without secondary impurities, while Bi doping effectively reduced the crystallite size, reaching a minimum of 13.6 nm at 0.4% Bi. SEM, EDX, and XPS analyses confirmed uniform morphology and successful incorporation of Bi^3+^ into the ZnFe_2_O_4_ lattice, accompanied by charge compensation involving partial Fe^3+^ → Fe^2+^ reduction without disturbing the spinel structure. Optical analysis showed a reduced band gap of 2.12 eV for the optimal sample, enhancing visible-light absorption. These modifications significantly improved photocatalytic activity, with the 0.4% Bi-ZnFe_2_O_4_ thin film achieving degradation efficiencies of 89% for rhodamine B and 75% for metronidazole within 120 min. Compared with reported ZnFe_2_O_4_-based photocatalysts, the optimized sample demonstrates competitive performance under visible light. Finally, the simplicity and scalability of spray pyrolysis make this approach promising for practical water treatment applications. Overall, 0.4% Bi doping is identified as the optimal concentration for enhancing the performance of ZnFe_2_O_4_ thin films for visible-light-driven photocatalysis.

## Supplementary Material



## Notes

Profilometer measurement of film thickness; SEM; XPS survey spectra; band gap energies (Eg) data; pseudo-first-order rate constants (*k*_1_); proposed photocatalytic mechanism; photocatalytic degradation efficiency reports (PDF)
