# Supplementary material for: Enhanced Visible-Light Photocatalytic Activity of Bi-Doped ZnFe2O4 Thin Films toward Rhodamine B and Metronidazole Degradation
Source: ACS Omega. 2026 Sep 28;11(40):60771–84. doi: 10.1021/acsomega.6c03278 (PMC13670255; doi:10.1021/acsomega.6c03278)
Supplement: Supplementary file 1 [file ao6c03278_si_001.pdf]

Supporting Information

Enhanced Visible-Light Photocatalytic Activity of Bi-doped $ZnFe_2O_4$ Thin Films Toward Rhodamine B and Metronidazole Degradation

Fatma Ezzahra Dhif [a*], Neila Jebbari[a], Enrique Rodriguez Castellon[b], Pankaj Kumar[d], Alberto Vomiero[c,d], Elisa Moretti[c], Najoua Turki-Kamoun[a,] and Kassa Belay Ibrahim[c*]

[a] Laboratory of Condensed Matter Physics, Faculty of Sciences, University of Tunis El Manar, 2092 Tunis, Tunisia.

[b] Departamento de Química Inorgánica, Cristalografía y Mineralogía, Facultad de Ciencias, Universidad de Málaga, Campus de Teatinos, Málaga 29071, Spain

[c] Department of Molecular Sciences and Nanosystems, Ca' Foscari University of Venice, Via Torino 155, 30172 Venezia Mestre, Italy

[d] Division of Materials Science, Department of Engineering Sciences and Mathematics, Luleå University of Technology, SE-97187 Luleå, Sweden

*E-mail: kassabelay.ibrahim@unive.it
dhiffatmaezzahra@gmail.com

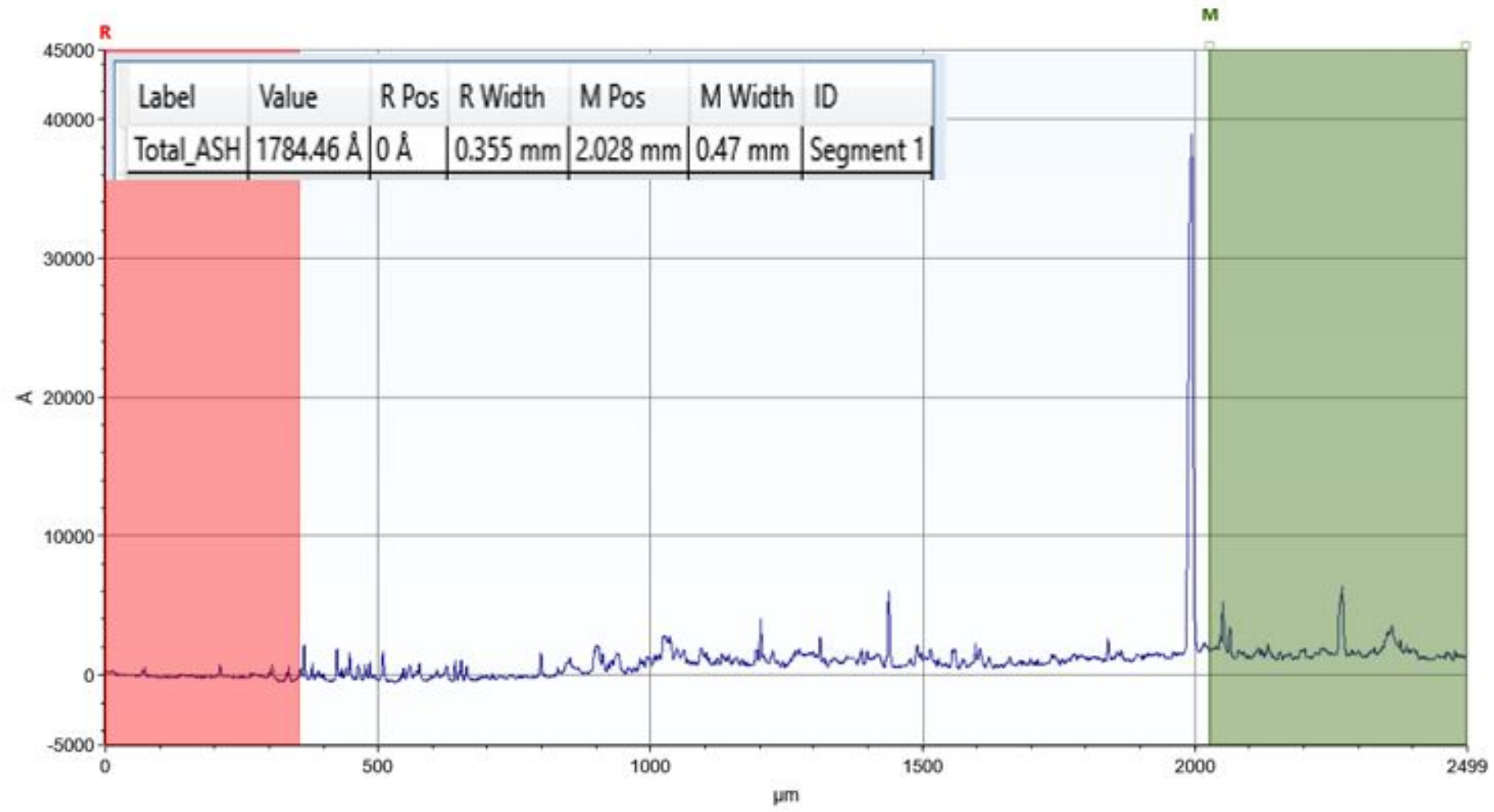

Figure S1: Profilometer Measurement of Film Thickness for 0.4%Bi-Doped $ZnFe_2O_4$ Thin Films

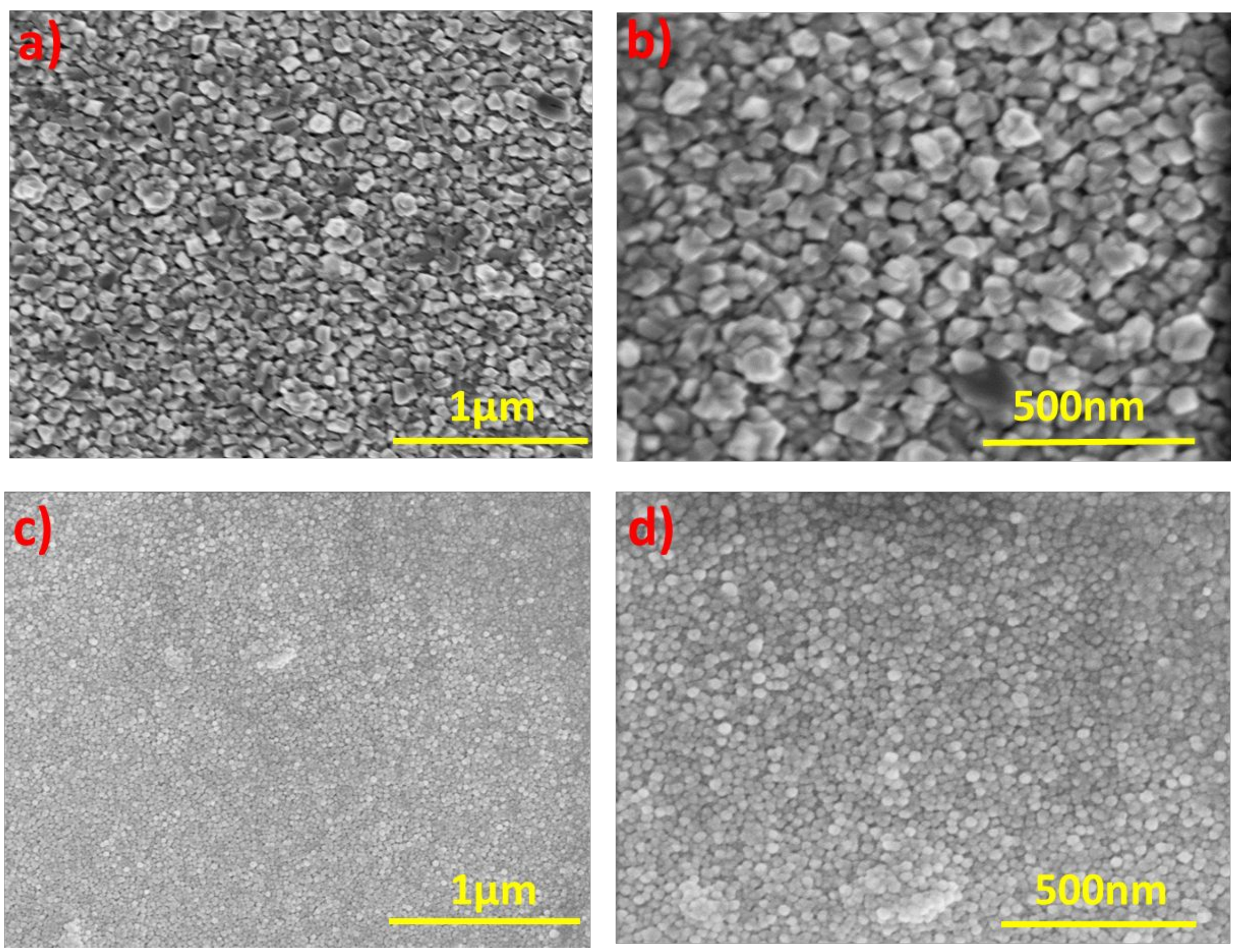

Figure S2: SEM images of (a-b) pristine $ZnFe_2O_4$ and (c-d) 0.4%Bi- $ZnFe_2O_4$ thin films.

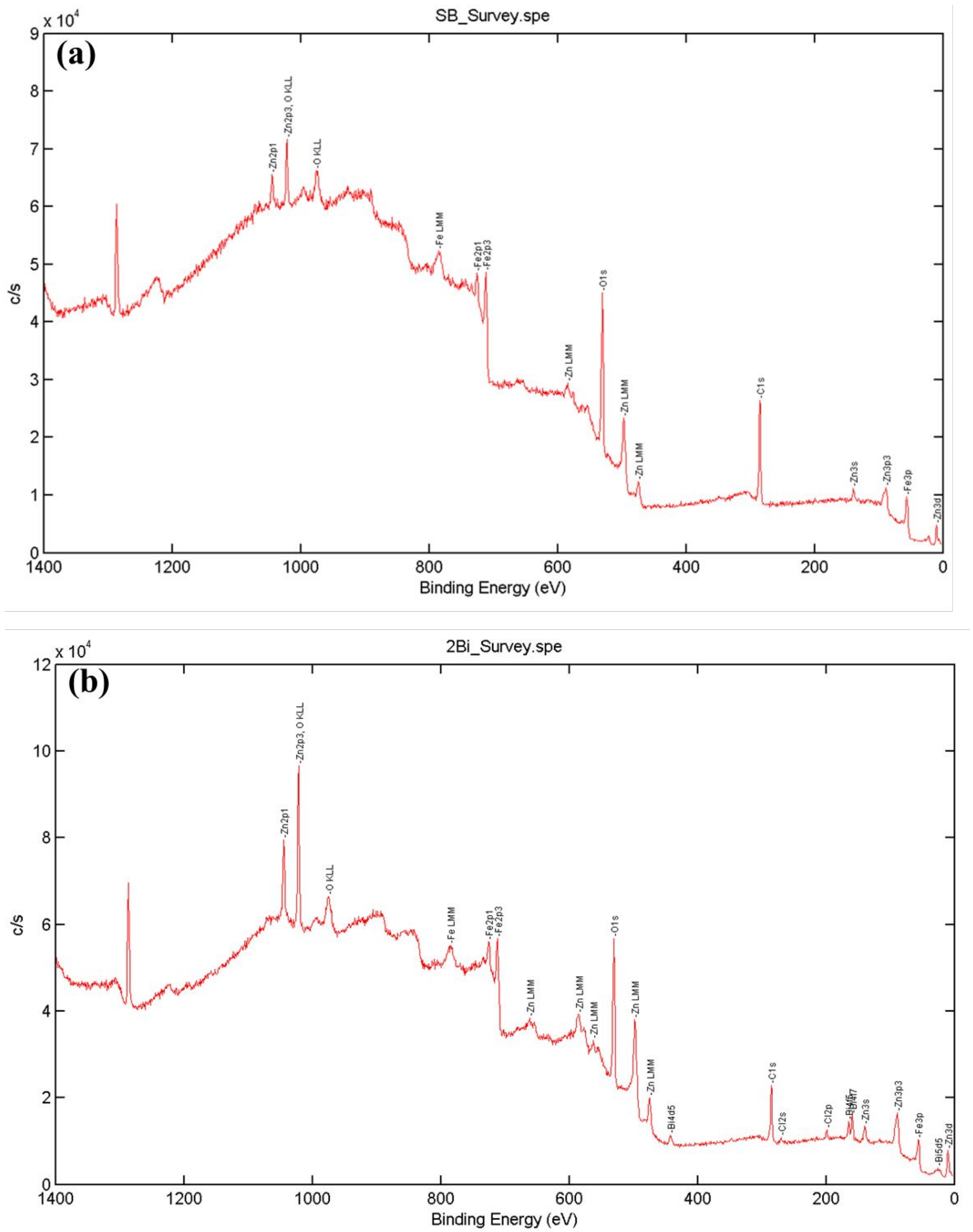

Figure S3: XPS survey spectra of (a) $ZnFe_2O_4$ and (b) 0.4% Bi- $ZnFe_2O_4$.

Table S1: Band gap energies (Eg) of undoped and Bi-doped $ZnFe_2O_4$thin films calculated from Tauc plots.

Sample	Band gap energy (eV)
**Undoped $ZnFe_2O_4$**	2.27
**0.2% Bi- $ZnFe_2O_4$**	2.25
**0.4% Bi-$ZnFe_2O_4$**	2.12
**0.6% Bi- $ZnFe_2O_4$**	2.15
**0.8% Bi- $ZnFe_2O_4$**	2.24

Table S2: Apparent pseudo–first-order rate constants (k_1), fitting error and corresponding R^2 values for MDZ photodegradation over the studied thin film samples.

Sample	Rate Constant (min^{-1})	Fitting error (±) (min^{-1})	Coefficient of Determination R^2
**Undoped $ZnFe_2O_4$**	0.00327	0.00031	0.97364
**0.4% Bi- $ZnFe_2O_4$**	0.01178	0.00144	0.95678

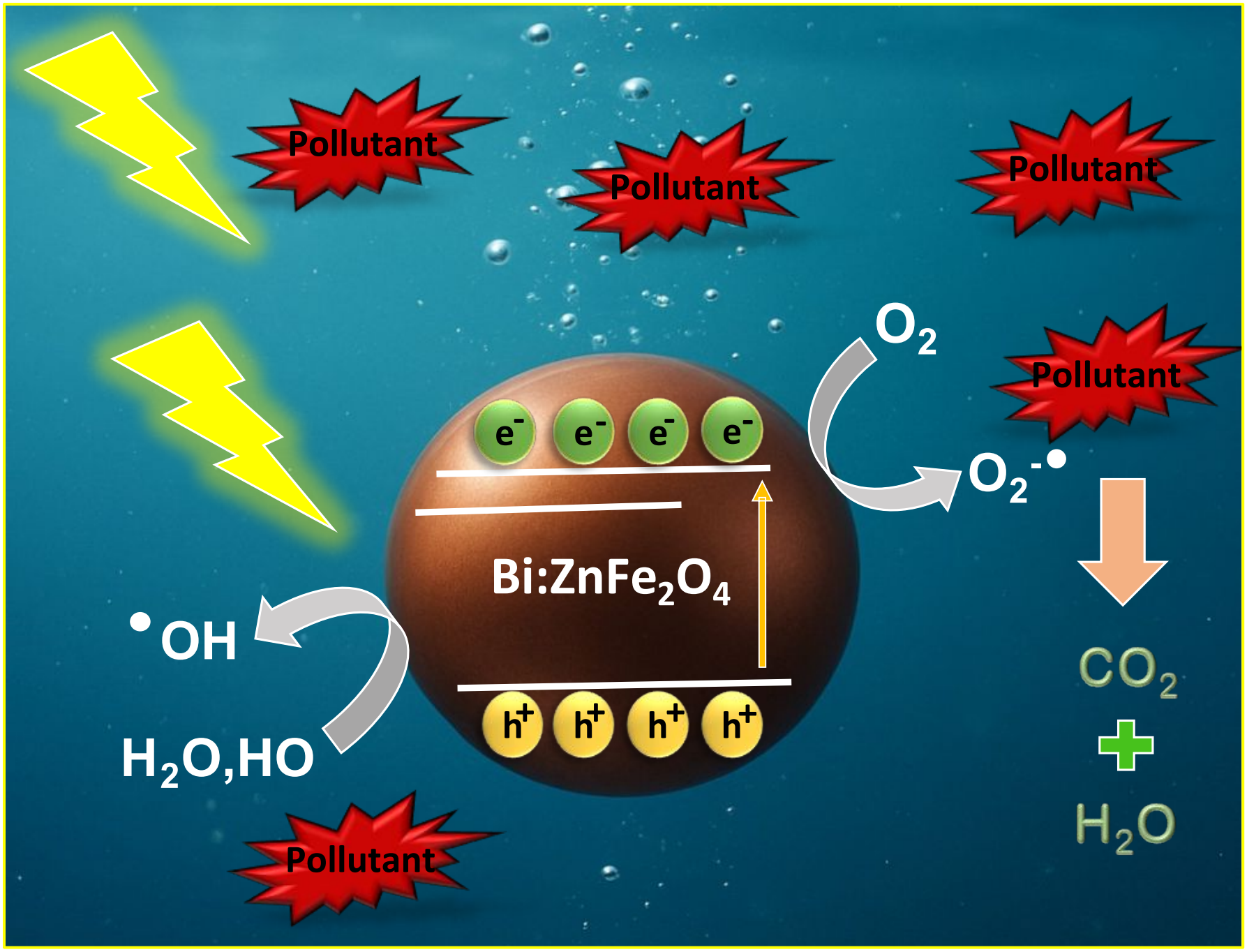

Figure S4: Proposed photocatalytic mechanism of Bi-doped $ZnFe_2O_4$ under visible-light irradiation.

Table S3: Photocatalytic degradation efficiencies of Bi-doped and other reported photocatalysts for the removal of organic pollutants under different irradiation conditions.

Photocatalysts	Pollutant	Irradiation Time (min)	Type of Irradiation	% Degradation	Ref.
Ce-doped $CuFe_2O_4$	Rhodamine b	120	Vis	88	[1]
Bi-doped SnS quantum dots	Crystal violet and Methylene Blue	120	Vis	95.08 and 89	[2]
Bi-doped ZnO	Methylene Blue	300	UV	65.976	[3]
$ZnO–ZnAl_2O_4$	Metronidazole	120	Sunlight	50.0	[4]
SBA-15/TiO_2		200	UV	87.70	[5]
Bi: $ZnFe_2O_4$	Rhodamine B and Metronidazole	120	Vis	89 and 75	This work

Reference:

[1] Keerthana, S. P., Yuvakkumar, R., Ravi, G., Pavithra, S., Thambidurai, M., Dang, C., & Velauthapillai, D. (2021). Pure and Ce-doped spinel $CuFe_2O_4$ photocatalysts for efficient RhB degradation. *Environmental Research*, *200*, 111528.

[2] Murugadoss, G., Venkatesh, N., Pandurengan, S., Thiruppathi, G., Sundararaj, P., & Gnanasekaran, L. (2025). Photocatalytic Degradation Using Bi-Doped SnS Quantum Dots and Phytotoxicity Evaluation of Treated Effluents Through Seed Germination. *Chemical Physics Impact*, 100944.

[3] Khumaeni, A., Jonathan, F., Alkian, I., Sugito, H., Nurhasanah, I., Ibrahim, R. K. R., & Julkapli, N. M. (2025). Direct synthesis of bismuth-doped ZnO nanoparticles in liquid precursor: A one-step pulsed laser ablation approach for enhanced photocatalytic dye removal. *Journal of Molecular Liquids*, 128054.

[4] Ghribi, F., Sehailia, M., Aoudjit, L., Touahra, F., Zioui, D., Boumechhour, A., ... & Benmaamar, Z. (2020). Solar-light promoted photodegradation of MDZ over ZnO-ZnAl2O4 heterojunction derived from 2D-layered double hydroxide structure. *Journal of Photochemistry and Photobiology A: Chemistry*, *397*, 112510.

[5] Sales, G. S., França, A. A., Cruz-Filho, J. F., Moraes, C. A., Neto, A. R. S., Sales, A. G., ... & Luz Jr, G. E. (2023). Photodegradation mechanism of MDZ on nanostructured material type SBA-15/TiO2. *Journal of Environmental Chemical Engineering*, *11*(5), 110335.
